# A Protocol to Extract a Specific Genomic Region from a Public Whole-Genome Database and Modify Analytical Bin Length for Population Genetic Studies

**DOI:** 10.3390/mps7040057

**Published:** 2024-07-27

**Authors:** Muhammad Shoaib Akhtar, Shoji Kawamura

**Affiliations:** Department of Integrated Biosciences, Graduate School of Frontier Sciences, The University of Tokyo, Kashiwa 277-8562, Chiba, Japan; kawamura@edu.k.u-tokyo.ac.jp

**Keywords:** population genetics, whole-genome sequencing, whole-exome sequencing, target capture, evolutionary studies

## Abstract

With the advent of “next-generation” sequencing and the continuous reduction in sequencing costs, an increasing amount of genomic data has emerged, such as whole-genome, whole-exome, and targeted sequencing data. These applications are popular not only in mega sequencing projects, such as the 1000 Genomes Project and UK BioBank, but also among individual researchers. Evolutionary genetic analyses, such as the dN/dS ratio and Tajima’s *D*, are demanded more and more for whole-genome-level population data. These analyses are often carried out under a uniform custom bin size across the genome. However, these analyses require subdivision of a genomic region into functional units, such as protein-coding regions, introns, and untranslated regions, and computing these genetic measures for large-scale data remains challenging. In a recent investigation, we successfully devised a method to address this issue. This method requires a multi-sample VCF file containing population data, a reference genome, target regions in the BED file, and a list of samples to be included in the analysis. Given that the targeted regions are extracted in a new VCF file, targeted population genetic analysis can be performed. We conducted Tajima’s D analysis using this approach on intact and pseudogenes, as well as non-coding regions.

## 1. Introduction

With the advent of “next-generation” sequencing (NGS) technologies and increased affordability through cost reductions, NGS data are emerging for many species [1,2] These include sequence assemblies from individuals to populations for many species [3,4,5]. For individual assemblies, data are usually stored and presented in FASTA format [6]. However, for population sequencing data, it is challenging to store, analyze, and interpret sequencing information using the FASTA format, particularly when multiple populations are included [7,8,9]. Other genomic data formats include the general feature format (GFF) and variant call format (VCF) [9,10]. The VCF format is commonly used nowadays because it stores the variation data of each sample along with the reference genome. This format was developed for the 1000 Genomes Project and can store haplotype information and variant annotations [11,12].

With the availability of population sequencing data, population genetic investigations have been accelerated for deciphering the effects of demography and natural selection on genetic variation. For example, Tajima’s *D*, Fu and Li’s G, F, and D, and Fay and Wu’s *H* are popular analytical methods of them [13,14,15]. Several tools to conduct these analyses for whole-genome data are available. Some of these measures are also equipped in some commonly available toolkits such as MEGA [16], pegas [17], PopGenome [18], VCF-kit [19], and VCFTools [9]. Most of these programs calculate these measures under a uniform custom bin size across the genome.

The uniform bin size occasionally spans a junction of a protein-coding (”coding” hereafter) exon and its adjacent non-coding region, such as an intron. Coding exon regions are more likely to be subject to natural selection than introns. In such a situation, the population genetic measure value at a junction bin could be compromised between these regions. One bin could encompass multiple exons and introns, and even two or more genes. Such generalized settings compromise the detection of natural selection.

It is important to calculate these measures separately using functional units. Analyzing targeted regions using the abovementioned tools remains a challenge because of uniform bin sizes. Alternative methods require making a consensus sequence (e.g., using bcftools consensus [20]) followed by alignment of those sequences using an alignment program (e.g., using Clustal W [21]) and then calculating these measures (e.g., using MEGA [22]). This approach is not only laborious but also time consuming and requires a large computing platform, especially in the case of large datasets. There are also tools that can detect natural selection in multi-sample VCF files, but they work under a uniform bin size. These tools include the VCF-kit and VCFtools [9,19]. Thus, there is a need to develop a method that can run selection tests on targeted genomic regions.

In this protocol, we describe a workflow to extract the targeted region of VCF files. A BED file and a list of samples were required to define the study population for analysis [23]. This workflow utilizes GATK SelectVariants and VCF-kit to extract targets and run a population genetic measure, respectively [19,24]. We successfully applied this protocol in our previous study to conduct Tajima’s *D* analysis of the target regions [25].

## 2. Protocol Design

**Figure 1:**
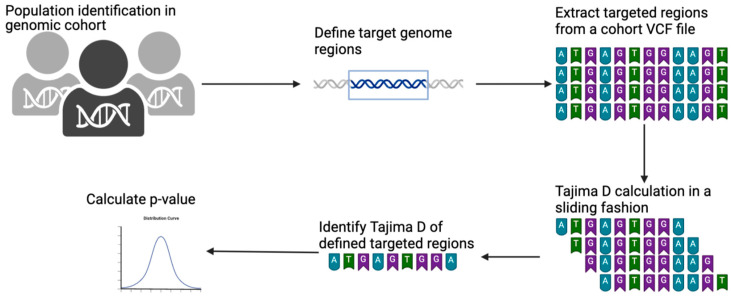
Schematic illustration of Tajima’s D calculation on a VCF file using a BED file. Protocols started from the identification of targeted populations and/or individuals in the genomic cohort. Targeted genomic regions were then defined by giving a BED file. Targeted regions were then extracted from a given VCF file. Tajima’s D was then calculated in sliding fashion, and an accurate targeted region was extracted.

This protocol involves extracting individuals or populations from a larger genome cohort in a multi-sample VCF file. Once extracted, the targeted genomic regions are defined using the chromosome number with the start and end coordinates of the reference genome. The targeted regions are defined using a BED file. The extraction of individuals and targeted genomic regions can be performed simultaneously. We used the GATK SelectVariants program for both the extraction of individuals and population purposes [24]. The extracted output VCF file can then be used for any genetic measure calculation. For example, we calculated Tajima’s *D* from this extracted VCF using the VCF-kit program. The VCF-kit is a program that can run Tajima’s *D* in sliding windows for a defined bin length [19]. We used a shell command to calculate the bin length for each targeted region and ran it in a sliding fashion. Once run, we used a grep command to pick up the real Tajima’s *D* of the targeted region. This design is illustrated in Figure 1.

## 3. Materials and Dependencies

To execute this method, the following items are necessary:

Reference genome data in FASTA format.

VCF: variant call format file of study samples containing genotype information for each variant.

BED file: browser extensible data format containing targeted genomic coordinate information for genetic measure calculation. A standard BED file contains chromosome/contig information and the start and end positions of the genomic interval.

List of samples: a text file containing a list of samples to be analyzed during the desired measure calculation. This option is helpful when samples representing many populations are included. This option can be utilized multiple times with different samples to obtain separate results for each population.

In addition, the following programs should be installed in the computing machine:

Genome Analysis Toolkit (GATK 4.0 or higher)
VCF-kit (0.2.9)
Python 3.0 or higher
UNIX shell (version 4.x)

## 4. Detailed Procedure

This workflow consisted of five main steps, as described below.

### 4.1. Step 1: Split the BED Files

This is the first step of the workflow and contains a heading entitled “#Split BED file line by line” in the script given in Supplementary File S1. This step is required only when more than one interval is provided in the BED file. In this step, a multiple-interval bed file is split into single-interval bed files. Each bed file contains only one interval.

### 4.2. Step 2: Define the Bin Size

Population genetic measures, such as Tajima’s *D*, are highly sensitive to differences in the number of segregating sites [13]. The addition or removal of a single base pair to or from the targeted region may lead to a significant difference. Therefore, highly accurate bin sizing that reflects the location of the coding regions in the genomic data is required for the input. In the second step, this workflow defines the bin size for genetic measurement calculation. This step refers to the “#Calculate bin size” in the script provided in Supplementary File S1. This is a simple awk command that subtracts the start position from the end position in the BED file and adds one. When subtraction is performed, the answer is one smaller than its bin size. For example, if we have one bin size of 1000 bp starting from 1 bp to 1000 bp, when we subtract first position 1 from position 1000, then the answer is 999 (1000 − 1 = 999). The 1000 bp here is arbitrary. One can choose any number based on their sequence length. To fix this difference, our protocol adds one to this step after subtraction is performed. Such missing nucleotide information can lead to false Tajima’s *D* results.

### 4.3. Step 3: Extract Target Regions and Individuals in the VCF File

This step is entitled “#Make a VCF for each BED file” in the script given in Supplementary File S1. The BED files made in Step 1 are used during this stage. GATK 4.0 or higher is required at this stage. GATK has several utilities for genomic data, but our script uses only the SelectVariants tool. This tool primarily requires a multi-sample VCF file accompanied by a BED file (generated in Step 1) to extract the targeted genome regions. If a multi-VCF file contains more than one population or if one wants to target only selected individuals, then a list of individuals to be included should also be provided. Such a file can be created using any text editor and saved with the .args suffix.

We recommend using only variants with the variant filter “PASS, ” biallelic, and exclude non-variant positions. Using only PASS-filtered variants will include variants that qualify all of the quality criteria applied to a VCF file. This means that only real variants will be included and any false variants will be excluded. Sometimes, the VCF file contains multiallelic single-nucleotide polymorphism (SNP) sites. When working with human genome data, we recommend using only biallelic SNPs and excluding multiallelic SNPs. The SelectVariants tool is designed to include all ploidy variants, either biallelic, triallelic, or any other allelic composition. However, this allelic composition can be manually set using the SelectVariants argument, “--restrict-alleles-to”. We recommend using only biallelic variants unless multiallelic variations have been confirmed through haplotyping and/or long-read sequencing. In such situations of confirmed multiallelic variation, this argument can be adjusted to the number of alleles. The last treatment that we recommend is to exclude nucleotide sites with no variation (exclude-non-variants) while maintaining sequence length. As population genetic measures only require variant sites, removing such sites leads to faster computation speed and the utilization of less memory.

Below, we show the running of the Tajima’s *D* workflow as an example. This Tajima’s *D* analysis was performed in our previously published paper [25]. Therefore, Tajima’s *D* can be replaced with any other genetic measure for which targeted regions are desired. We used the VCF-kit program to calculate Tajima’s *D*, and all arguments are described in detail in the next step.

### 4.4. Step 4: Run Tajima’s D

**Figure 2:**
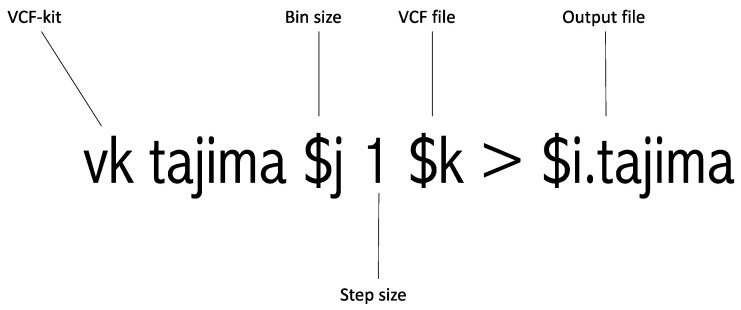
Graphical explanation of running the Tajima’s D command.

This step is entitled “#Run Tajima’s D” in Supplementary File S1. This is the most crucial step in this pipeline as it involves running Tajima’s *D*. This step depends on the VCF-kit and utilizes the VCF file generated in Step 3 as an input file. Moreover, the bin size calculated in Step 2 is incorporated here. This step is illustrated in Figure 2. Python 3.0 or higher should be installed on the computing platform for this step. The output of each targeted region contains the “.tajima” suffix. The VCF-kit is an established tool for Tajima’s *D* analysis, but it is designed for whole-genome analysis. This tool conducts Tajima’s *D* and returns the computed Tajima’s *D* value. Therefore, we added a sliding of 1 bp to the command. This 1 bp sliding runs Tajima’s *D* from the starting to the end position every 1 bp. For example, if one adds a 1 bp sliding to the 1000 bp targeted region, it runs Tajima’s *D* from 1 to 1000 bp, 2 to 1001 bp, 3 to 1002 bp, 4 to 1003 bp, etc., as shown in Figure 1 earlier. Once this analysis is completed, the targeted region of interest can be easily extracted. This extraction procedure is described in the following step.

### 4.5. Step 5: Extract the Targeted Region

Once Tajima’s *D* is run, the next step is to extract regions of interest from the .tajima output file. This is the first output file with the Tajima’s *D* analysis. Because this file contains many genomic regions, extracting the correct genomic region with Tajima’s *D* is crucial. Our script part “#extract target regions” in Supplementary File S1 can perform this successfully. The output file “tajima” contains the start and end positions of the targeted region.

Our protocol utilizes two approaches for extraction. The first approach given in the script was specifically used for targeted sequencing experiments. In targeted sequencing experiments, the targeted regions have the highest number of variants. The output file “tajima” contains the number of variants in column 4. Therefore, our script first extracts column 4 and sorts it from highest to lowest for the number of variants. Based on the highest number of variants, it simply utilizes the Linux grep command and extracts the Tajima’s *D* of the region. Alternatively, we utilized a second approach that directly utilized the grep command system. In this manner, the grep command is piped as follows:

grep “start position” file.tajima | grep “end position” > output_target.tajima

This second approach is best when working with whole-genome sequencing data, for example, when working with 1000 Genomes Project data, etc., because it is very specific for start and end positions. The start and end positions can be simply used from the starting BED file.

The script for this workflow is provided in Supplementary Files S1 and S2. The script for extracting targeted regions and running Tajima’s D is given in Supplementary File S1, while running Tajima’s D of the targeted regions on a WGS VCF can be performed using Supplementary File S2. Readers can replicate this script either by running it once or step by step.

## 5. Results

We tested this protocol using in-house targeted capture sequencing data of 69 anonymized Japanese individuals, including 403 human autosomal olfactory receptor (OR) intact genes, 99 human OR pseudogenes, and 81 autosomal neutral references (NRs) [25]. Intact human OR genes are known to have an open reading frame of at least 250 amino acids without any disruption, while sequences with an open reading frame shorter than 250 amino acids are regarded as pseudogenes [25,26]. NRs are single-copy non-protein-coding sequences that are at least 0.2 centimorgan away from a protein-coding region with a minimum length of 1000 bp [25]. We determined these neutral references using neutral region explorer software [27]. We were able to successfully conduct this pipeline for Tajima’s *D* calculation. However, readers can replicate this pipeline to calculate other genetic measures, too. Targeted VCF files generated during step 3 can be utilized for this purpose. VCF files are good to use with the VCF-kit and VCFtools or can be imported into R or Python to perform any kind of downstream analysis specific to targeted regions.

Once Tajima’s *D* or other population genetic measures are calculated, results can be statistically verified using a parametric independent sample t-test or a non-parametric equivalent and interpreted accordingly. We ran this program on a Linux Ubuntu 22.04.4 LTS (64 bit) installed on an Intel^®^ Core^TM^i9-10900K CPU @ 3.70GHz × 20 machine with 32.0 GB memory and 12.3TB disk capacity. Though it was a large cohort for which we conducted this analysis in our previous paper, we picked up a single gene of 943 nucleotides to measure wall time and memory usage. It took only 0.01 min to extract the targeted gene into a multi-sample VCF file. However, it took 5 min and 16 s to conduct the entire analysis using 1% CPU. To validate these results, we also conducted this analysis on a MacBook Pro with an Apple M2 (2023) chip with 24 GB memory and 994.66GB disk capacity. Although the VCF extraction time remained the same, the Tajima’s D calculation time was shortened to 3 min and 51 s while a 99% CPU was used. Thus, our protocol is an efficient way to extract the targeted regions from a large VCF file. However, computing Tajima’s D is a computationally intensive procedure, and higher CPU usage can reduce the processing time.

## 6. Discussion

We successfully performed the current protocol. The protocol was aimed at calculating Tajima’s *D* for the targeted regions in a multi-sample VCF file. This protocol utilizes GATK and VCF-kit utilities. The GATK SelectVariants tool was used to extract the targeted regions. GATK pipelines and tools have been proven to process NGS reads and manipulate VCF files [24,28,29,30,31]. GATK pipelines and tools have already been used in large-scale genome projects, including the 1000 Genomes Project [11,12]. In recent years, many human genomic studies published have cited GATK pipelines, resulting in over 40,000 citations to date. In our previous paper, we compared variant sites and SNP density among protein-coding and non-protein-coding regions when NGS reads were processed using GATK. We also found this to be an efficient method for variant calling and copy number variation analysis [25].

Once the targeted region was extracted, our protocol was dependent on the VCF-kit for Tajima’s *D* calculation. The VCF-kit by itself is an established program; therefore, we do not discuss the efficiency of its calculation [19] as our protocol is more focused on providing a solution for the calculation of targeted regions in a VCF file. Both major programs, GATK and VCF-kit, are established and authenticated tools. Several studies have established the authenticity of these tools [19,24,28,29,30,31]. Therefore, this is beyond the scope of the current protocol. However, scientists who want to test other genetic measures can use the targeted VCF files generated during step 3, and any form of downstream analysis can be performed. VCF files have the advantage of compatibility with several programs and programming languages, including R and Python. These extracted VCF files can be used for almost any genetic analysis with accurate targeted regions. A VCF file can be imported into R or Python, for example, the vcfR program can be used to read a VCF file in R [32].

We successfully computed Tajima’s *D* values for the human OR gene family and neutral genomic regions. This workflow successfully extracted accurate desired regions of OR genes. This extraction helped us to obtain the Tajima’s *D* value of each target with confidence. This value was calculated for a specific gene length for each gene and produced an accurate result. For population geneticists looking into genomic data, it is important to accurately examine phenomena in their targeted regions, and our workflow performed this process successfully. Anyone who wants to extract target-specific genomic lengths can use this workflow for nearly any genetic measure calculation.

In addition to these merits, this protocol has some limitations too. The first limitation is the protocol’s dependency on GATK and VCF-kit. These tools must be installed and configured on a computer. This dependence limits the applicability of the current protocol’s portability. Our protocol is based on GATK4; therefore, older GATK versions may produce different results. Furthermore, both of these dependencies can only be installed on a UNIX or Mac shell and cannot be used on Microsoft Windows. Moreover, the protocol requires an understanding of the UNIX command-line interface. Another limitation is minimal error checks in the protocol. The protocol checks whether all five arguments are given and produces an error if fewer than five arguments are given. The missing arguments were also identified. Out of these five arguments, four were files. Finally, the protocol processes files sequentially and may require longer processing times when working with big genomic data.

In conclusion, this workflow protocol can accurately extract genomic regions from large VCF files, genomic datasets, or targeted capture experiments. The extracted VCF files can be used to compute a number of population genetic measures. We successfully demonstrated Tajima’s *D* computation. Output VCF files can also be imported into R and Python utilities for almost any type of computational analysis. This protocol can be applied to any set of genes within a species when all of the desired information is available.

## Data Availability

Data used in this protocol can be found in our previously published original research article accessible at https://doi.org/10.1537/ase.211024. This pipeline can be accessed at https://github.com/xoaib4/vcf2tajima.

## Supplementary Materials

The following supporting information can be downloaded at: https://www.mdpi.com/article/10.3390/mps7040057/s1, Supplementary File 1: File S1; Supplementary File 2: File S2.
