# Supplementary material for: A Protocol to Extract a Specific Genomic Region from a Public Whole-Genome Database and Modify Analytical Bin Length for Population Genetic Studies
Source: Methods Protoc. 2024 Jul 27;7(4):57. doi: 10.3390/mps7040057 (PMC11357298; doi:10.3390/mps7040057)
Supplement: Supplementary file 1 [file mps-07-00057-s001.zip › Supplementary File S1.pdf]

```
########################
##Supplementary File S1##
########################



#!/bin/bash

# Usage: ./vcf2tajima_target.sh targets.bed Reference.fasta variants.vcf names.args output

# Define input parameters
TARGETS_BED=$1
REFERENCE_FASTA=$2
VARIANTS_VCF=$3
NAMES_ARGS=$4
OUTPUT=$5

# Function to print usage
usage() {
   echo "Usage: $0 targets.bed Reference.fasta variants.vcf names.args output"
   echo "  targets.bed  : Path to the BED file containing target regions"
   echo "  Reference.fasta : Path to the reference FASTA file"
   echo "  variants.vcf : Path to the VCF file with variants"
   echo "  names.args   : Path to the sample names arguments file"
   echo "  output       : Path to the output file with .tajimaD suffix"
   exit 1
}

# Check if the correct number of arguments is provided
if [ "$#" -ne 5 ]; then
   echo "Error: Incorrect number of arguments provided."
   usage
fi

# Check if the necessary files exist
if [ ! -f "$TARGETS_BED" ]; then
   echo "Error: $TARGETS_BED not found!"
   usage
fi

if [ ! -f "$REFERENCE_FASTA" ]; then
   echo "Error: $REFERENCE_FASTA not found!"
   usage
fi

if [ ! -f "$VARIANTS_VCF" ]; then
   echo "Error: $VARIANTS_VCF not found!"
   usage
fi

if [ ! -f "$NAMES_ARGS" ]; then
```

```
    echo "Error: $NAMES_ARGS not found!"
    usage
fi

# List files in the current directory
ls

mkdir temp
cd temp

# Split BED file line by line, each output file contains one line from the input BED file
split -l 1 ../"$TARGETS_BED" --additional-suffix=.bed

# Loop through each split BED file
for i in *.bed; do
    echo "Processing BED file: $i"

    # Calculate bin size
    j=$(awk '{print $3 - $2 + 1}' "$i")
    echo "Bin size: $j"

    # Make a VCF for each BED file
    gatk SelectVariants -R ../"$REFERENCE_FASTA" -V ../"$VARIANTS_VCF" -L "$i" -O "$i.vcf" --exclude-filtered --exclude-non-variants --restrict-alleles-to BIALLELIC --sample-name ../"$NAMES_ARGS"

    # Loop through each VCF file
    for k in *.vcf; do
        echo "Processing VCF file: $k"

        # Run Tajima's D
        vk tajima "$j" 1 "$k" > "$i.tajima"

        # Extract target regions
        for l in *.tajima; do
            echo "Processing Tajima D file: $l"

            m=$(awk '{print $4}' "$l" | grep -Eo '[0-9]+' | sort -rn | head -n 1)
            echo "Maximum value: $m"

            grep -w "$m" "$l" > "${i}_cds.tajimaD"

            # Append results to the output file
            cat "${i}_cds.tajimaD" >> "../${OUTPUT}.tajimaD"

            # List files
            ls
        done
    done
done

# Clean up temporary directory
```

```
cd ..
rm -r temp
```
